# Insights into brain tumor diagnosis: exploring *in situ* hybridization techniques

**DOI:** 10.3389/fneur.2024.1393572

**Published:** 2024-07-03

**Authors:** E. D. Namiot, G. M. Zembatov, P. P. Tregub

**Affiliations:** ^1^Department of Pathophysiology, First Moscow State Medical University (Sechenov University), Moscow, Russia; ^2^Brain Research Department, Federal State Scientific Center of Neurology, Moscow, Russia; ^3^Scientific and Educational Resource Center, Innovative Technologies of Immunophenotyping, Digital Spatial Profiling and Ultrastructural Analysis, Peoples' Friendship University of Russia (RUDN University), Moscow, Russia

**Keywords:** *in situ* hybridization, FISH, glioblastoma, oligodendroglioma, meningioma, ependymoma, medulloblastoma, pituitary adenoma

## Abstract

**Objectives:**

Diagnosing brain tumors is critical due to their complex nature. This review explores the potential of *in situ* hybridization for diagnosing brain neoplasms, examining their attributes and applications in neurology and oncology.

**Methods:**

The review surveys literature and cross-references findings with the OMIM database, examining 513 records. It pinpoints mutations suitable for *in situ* hybridization and identifies common chromosomal and gene anomalies in brain tumors. Emphasis is placed on mutations’ clinical implications, including prognosis and drug sensitivity.

**Results:**

Amplifications in EGFR, MDM2, and MDM4, along with Y chromosome loss, chromosome 7 polysomy, and deletions of PTEN, CDKN2/p16, TP53, and DMBT1, correlate with poor prognosis in glioma patients. Protective genetic changes in glioma include increased expression of ADGRB3/1, IL12B, DYRKA1, VEGFC, LRRC4, and BMP4. Elevated MMP24 expression worsens prognosis in glioma, oligodendroglioma, and meningioma patients. Meningioma exhibits common chromosomal anomalies like loss of chromosomes 1, 9, 17, and 22, with specific genes implicated in their development. Main occurrences in medulloblastoma include the formation of isochromosome 17q and SHH signaling pathway disruption. Increased expression of BARHL1 is associated with prolonged survival. Adenomas mutations were reviewed with a focus on adenoma-carcinoma transition and different subtypes, with MMP9 identified as the main metalloprotease implicated in tumor progression.

**Discussion:**

Molecular-genetic diagnostics for common brain tumors involve diverse genetic anomalies. *In situ* hybridization shows promise for diagnosing and prognosticating tumors. Detecting tumor-specific alterations is vital for prognosis and treatment. However, many mutations require other methods, hindering *in situ* hybridization from becoming the primary diagnostic method.

## Introduction

The importance of diagnosing brain tumors is underscored by the severity of their clinical presentations and the complexities involved in treating neoplasms within this region (1). It’s crucial to note the continued absence of effective therapeutic strategies for the most prevalent types of brain tumors (2, 3). Early diagnosis and molecular-genetic profiling of tumors are emerging as promising avenues for developing treatment modalities (4, 5).

The evolution of molecular-genetic techniques has significantly bolstered our capacity to prognosticate tumor outcomes and evaluate the propensity for tumor development (6–9). Within this domain, *in situ* hybridization methods, encompassing both fluorescent and chromogenic variants, play a pivotal role in tumor interrogation (10). These methodologies diverge primarily in signal detection mechanisms and sensitivity, with fluorescent hybridization being particularly salient in brain tumor diagnosis (11, 12). In the realm of neurobiology, the emergence of probes targeting mutations in key genes such as c-myc, EGFR, and topoisomerase IIa offers profound insights into brain tumor pathogenesis (13–16). These advancements hold promise for improving both diagnostic accuracy and treatment strategies for brain tumors.

The principle of *in situ* hybridization (ISH) relies on the interaction between labeled nucleotide probes and target RNA/DNA sequences (17–19). Generally, the process involves several steps: preparing the tissue or cells (such as cell pellets or paraffin-embedded tissues), preparing specific probes (often commercially available for routine diagnostics), and finally, the hybridization and visualization steps (18). Each of these steps has its own limitations. For example, fluorescent probes used in FISH (fluorescence *in situ* hybridization) analysis can only detect deletions up to 200 kb, leaving smaller genomic changes undetected (18, 20). Commercial probes typically come with detailed information, including the target gene, probe localization, and the specific protocol to be followed (18). Probe design is an area of ongoing research, with recent advancements such as improved detection of amyloid-β peptides in Alzheimer’s disease (21, 22). The duration of ISH analysis can vary significantly, with hybridization alone taking place overnight (18). Consequently, the entire analysis process can take several days.

In the field of genetic diagnostics, *in situ* hybridization techniques are indispensable for probing genetic material within cells without compromising tissue integrity (17). Widely embraced in medical practice, they offer precise detection of genetic variations (23). Fluorescence *In Situ* Hybridization (FISH) analysis is one of many possible options, enabling targeted hybridization during cellular division (24). Additionally, the advent of two-color chromogenic hybridization has further broadened the scope of genetic analysis (14, 25). These techniques utilize probes with complementary sequences and fluorescent tags for visualization, facilitating concurrent examination of multiple genetic targets (26–29). While *in situ* hybridization typically targets cells in the resting phase, its application to dividing cells occasionally enhances result clarity (30–32). Presently, gene-or location-specific probes are favored for their precision and can be tailored using DNA libraries (33, 34). These advancements hold promise for deepening our understanding of cellular genetics.

It’s worth highlighting that visualizing *in situ* hybridization results enables the application of diverse microscopic techniques for 3D signal visualization within tissues (35). Modern technologies such as FISHQuant, coupled with optimizations in analysis steps utilizing novel buffering systems to enhance tissue sample stability, are gaining widespread adoption (36). The stability of probes is contingent on various factors; for instance, DNA probes are generally deemed more resilient than mRNA, whereas microRNA exhibits remarkable stability (26).

Hybridization techniques provide insight not only into DNA but also RNA sequences simultaneously (13). When examining mRNA, chromogenic hybridization is a common choice, utilizing dioxigenin as a marker detectable through specific peroxidases (37–39). However, this method lacks the precision required for chromosomal analysis, essential for diagnosing chromosomal mutations—a prevalent cause of brain tumors. In neurogenetics, *in situ* hybridization can uncover anomalies such as microdeletions (indicating the absence of signal on one chromosome copy), translocations (evidenced by signals from a gene on one chromosome to another), and aneuploidies (revealing changes in chromosome) (16, 18). These genetic aberrations play a critical role in diagnosing various types of brain tumors (40).

Despite the promising potential of *in situ* hybridization techniques in tumor diagnosis, their application to brain tumors is hampered by the lack of comprehensive mutation data characterizing the molecular-genetic profile of these tumors. Thus, our review aimed to scrutinize literature sources delineating diagnostic features identified through *in situ* hybridization for profiling the most prevalent brain tumors. We then cross-referenced these findings with the Online Mendelian Inheritance in Man (OMIM) database, examining 513 records to pinpoint mutations suitable for *in situ* hybridization methods. Our analysis focused on mutations in genes with well-established molecular bases, excluding those associated with syndromes featuring multiple tumors, such as MSH6 and MLH1. Therefore, we included only the genes marked with an asterisk (*) and a plus sign (+) on the OMIM website, as all other entries (e.g., those with # and % symbols) refer solely to phenotype descriptions and do not represent specific loci.

### Astrocytomas and oligodendrogliomas

Glioblastomas, classified as Grade IV malignancies, exhibit the poorest prognosis among central nervous system tumors, with a median survival of only 15 months (40, 41). These tumors can be stratified into two distinct subgroups. The first subgroup, predominant in individuals over 60, is characterized by EGFR amplification, suggesting an unfavorable prognosis. Conversely, the second subgroup, more prevalent in younger patients, presents with a protracted disease course and is associated with mutations in the p53 transcription factor, a critical regulator of the cell cycle (42, 43). Notably, literature indicates that FISH hybridization does not confer significant advantages in detecting mutations within this gene during mitotic recombination (44). However, remarkable advancements have been made in the analysis of EGFR gene amplification, offering promising diagnostic avenues for anaplastic oligodendrogliomas and small cell glioblastomas. This advancement is particularly noteworthy considering the challenges posed by distinguishing these tumors solely through traditional histological methods. Furthermore, an intriguing aspect lies in the co-deletion of 1p/19q, playing a pivotal role in diagnosing anaplastic oligodendrogliomas and exhibiting notable sensitivity to chemotherapy (24).

Given the significant mortality rate attributed to glioblastoma, there is a particular interest in identifying genes associated with the risk of the most unfavorable prognosis. Using the FISH method, researchers have identified monosomies of chromosome 10q, frequently accompanied by trisomies of chromosome 7 (45). These genetic alterations, along with mutations or loss of the PTEN gene, are strongly linked to extremely low survival rates. However, despite this correlation, the method fails to distinguish between primary and secondary subgroups of glioblastomas (46). In a notable study, researchers observed that the transition from astrocytomas to glioblastomas is associated with sequential occurrences of trisomy of chromosome 7 and monosomy of chromosome 10. However, individually, these genetic aberrations lack diagnostic significance. Similar trends were observed for sex chromosomes, with the FISH method revealing a disomy of the X chromosome, often coupled with the absence of the Y chromosome, detected in 71% of primary glioblastoma samples (47).

Mutations in genes such as PTEN, DMBT1, CDK4, and the deletion of the tumor suppressor gene p16 have been identified in glioblastomas (48–53). In a study conducted by Koshiyama et al. (48), which involved 40 glioblastoma patients, FISH analysis revealed monosomy of chromosome 10 in 52.5% of cases, polysomy of chromosome 7 in 50%, and PTEN gene deletion in 35% of cases, all of which were associated with an unfavorable prognosis. Loss of p16 expression has been proposed as a prognostic factor in glioblastoma patients, especially when combined with IDH mutations (49). While glioblastomas with wild-type IDH lack prognostic value, other studies suggest a correlation between p16 deletion and increased chemotherapy sensitivity (49, 50).

Mutations in the DMBT1 gene, situated on chromosome 10, have also been associated with a poorer prognosis. Notably, most studies rely on PCR/qPCR techniques to detect mutations in this gene (51). Furthermore, while the FISH method is capable of detecting only monosomies for chromosome 10 mutations commonly observed in glioblastomas, microsatellite analysis proves more effective in identifying other variants (51). Another limitation of FISH is its inability to assess methylation status, crucial in genes like MGMT, which correlates with a more favorable chemotherapy outcome (54). For further insights, Table 1 offers an overview of the target genes analyzed in glioblastoma cells using the FISH technique.

**Table 1:** Mutations identified in glioblastoma cells using the FISH method.

Gene / chromosome	Prognosis	Additional information	References
Amplifications			
PDGFR	Does not affect prognosis.	Primary glioblastoma.*	(49)
EGFR	Poor (older than 60 years)**	Primary glioblastoma.	(27)
MDM2	Poor prognosis.	Resistance to EGFR-TKIs.	(47)
MDM4	Poor prognosis.	Resistance to EGFR-TKIs.	(47)
KDR	Does not affect prognosis.	More often with PDGFRA.	(50)
CDK4	Less resistance to bevacizumab.	Infiltration by immunosuppressive macrophages.	(51, 52)
KIT	Does not affect prognosis.	More common in individuals younger than 60 years old.	(49, 50)
VEGFR2	Does not affect prognosis.	Primary glioblastoma.*	(49)
Monosomies / chromosome losses			
Y chromosome	Poor prognosis.	Does not differentiate between primary and secondary glioblastomas.	(41, 53)
Chromosome 10	Does not affect prognosis.	Precedes trisomy of chromosome 7.	(41)
Polysomies			
X chromosome	Does not affect prognosis.	Inactivation in healthy women increases the risk of glioblastoma.	(41, 54)
Chromosome 7	Poor prognosis.	It can be used for diagnosis.***	(55)
Deletions			
PTEN	Poor prognosis.	When pTERT mutation occurs, it is often accompanied by EGFR amplification.	(41, 56)
CDKN2/p16	Poor prognosis.	Increased sensitivity to antimetabolites.	(39)
TP53	Poor prognosis.	Most commonly missense mutations.	(18)
1p/19q	For anaplastic gliomas, the prognosis is favorable, while for glioblastomas, the significance remains unclear.	The preferred treatment includes procarbazine, lomustine, and vincristine.	(55, 57)
DMBT1	Poor prognosis.	The preferred method is PCR/qPCR.	(45)

*These were also detected in secondary glioblastomas.

**The prognosis was better for individuals under 60 years old.

***Alongside EGFR amplification, monosomy of chromosome 10, mutations in pTERT, in the absence of typical histological features.

Despite its significance, FISH analysis encounters certain limitations, particularly in diagnosing pilocytic astrocytomas. This is due to their frequent association with neurofibromatosis type 1, characterized by the loss of NF1 gene expression (58). Detecting this deletion using fluorescent hybridization becomes practically impossible (59, 60). However, it’s worth noting that this limitation does not preclude the possibility of sporadic forms of astrocytomas, which can be examined using the FISH method provided there are relevant mutations (61). Thus, despite its limitations, FISH remains a valuable tool in diagnostics, offering insights into the molecular-genetic profile of tumors.

**Figure 1:**
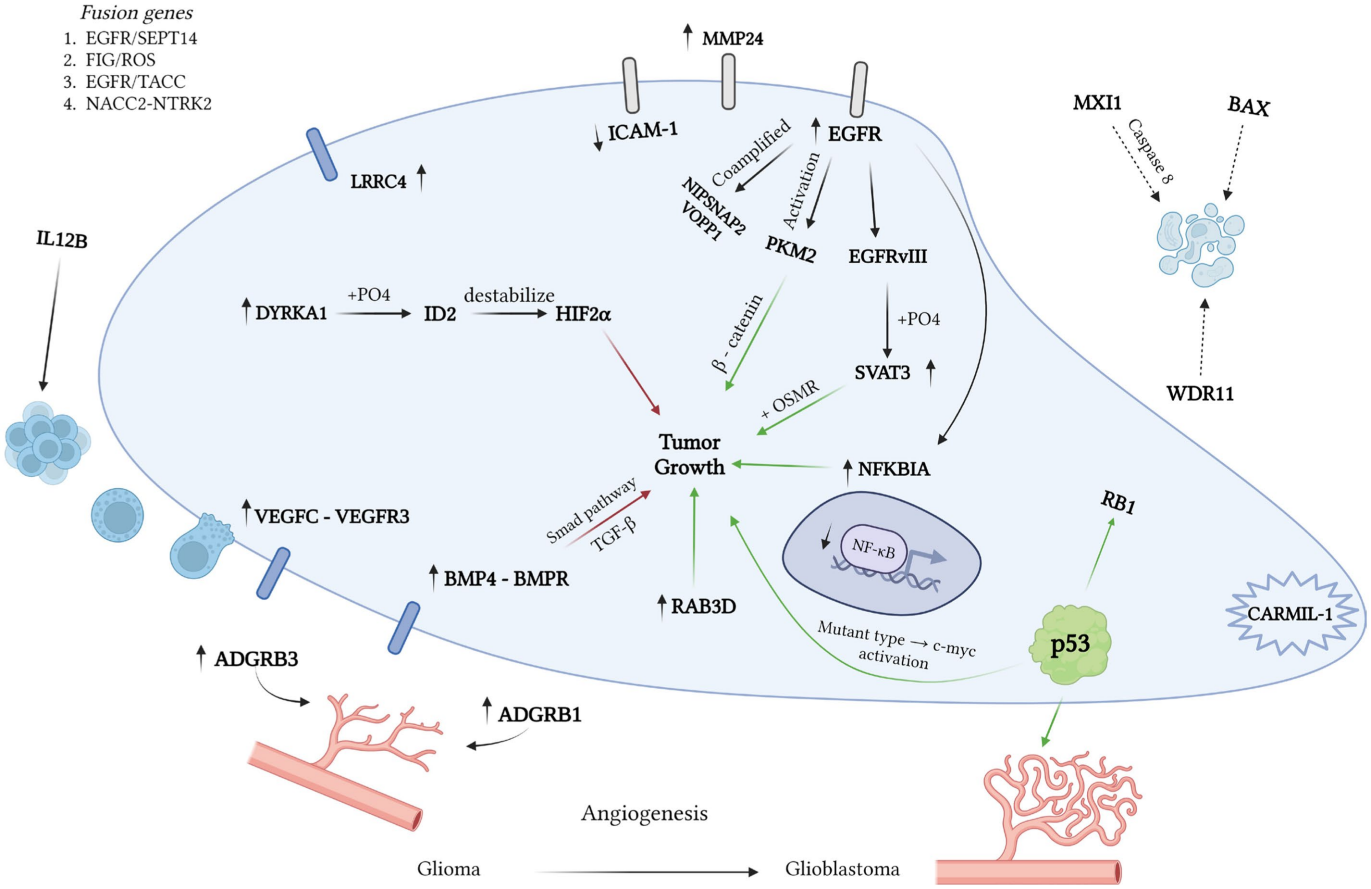
Various mutations influence the pathogenesis of glioblastoma, with green arrows denoting a positive effect and red arrows indicating a negative one. Using the OMIM database, we identified 265 records containing “glioblastoma” and “+glioblastoma.” Our analysis unveiled mutations with a positive prognosis linked to immune response activation. For instance, VEGFC expression enhances prognosis by fostering the binding of CD8 T cells to tumor cells (OMIM 601528). However, the glioblastoma microenvironment suppresses the immune response via high IL12 expression (OMIM 161561). LRRC4 and BMP4 expression correlate with smaller tumor size and slower growth (OMIM 610486; OMIM 112262). EGFR mutations, including hybrid genes EGFR/SEPT14 and EGFR/TACC (OMIM 131550; OMIM 612140), play a pivotal role in glioblastoma development. The formation of the mutant EGFRvIII variant results in increased SVAT3 expression and active progression (OMIM 601743). The SVAT3/OSMR complex portends a negative prognosis. Deletions in EGFR exons 2–7 and amplification in the EGFR gene are also critical (OMIM 131550). RAB3D is associated with low survival rates. Glioblastoma’s uncontrolled division stems from tyrosine kinase activation. The hybrid FIG/ROS gene and mutations in FGFR1 are linked to tyrosine kinase activation. Deletions in NFKBIA, NF1, and TRIM8, along with loss of heterozygosity in growth-suppressor genes (WDR11, BAX, MXI1), are associated with low survival rates. Co-amplification of NIPSNAP2 and VOPP1 accelerates tumor growth and worsens prognosis (OMIM 603004; OMIM 611915). Angiogenesis plays a crucial role in tumor growth, often stimulated by p53 gene mutation in high-grade gliomas. In benign tumors, neo-vascularization is inhibited by ADGRB1 expression (OMIM 602682).

Using the OMIM database, we examined 265 records containing “glioblastoma/+glioblastoma” fragments and selected mutations suitable for detection through hybridization methods (Figure 1). Throughout the database analysis, we identified mutations/changes with positive prognostic implications, as well as those linked to glioblastoma development or bearing a negative prognosis. Notably, a substantial portion of changes associated with a favorable prognosis involved immune response activation in response to tumor growth. For instance, heightened ectopic expression of VEGF-C facilitated the binding of CD8+ receptors on T-lymphocytes to tumor cells (OMIM 601528). However, the glioblastoma microenvironment, alongside tumor cells themselves, often exhibits immunosuppressive effects, which can be counteracted by elevated IL-12 cytokine expression (OMIM 161561). Furthermore, decreased ICAM-1 receptor expression correlated with reduced tumor sensitivity to cytotoxic lymphocytes (OMIM 147840). Conversely, elevated LRRC4 and BMP4 expression correlated with smaller tumor sizes and slower growth rates (OMIM 610486; OMIM 112262). It’s worth noting that while most researchers employ PCR for expression analysis, the possibility of utilizing *in situ* hybridization methods for the same purposes remains open.

One of the crucial steps in the pathogenesis of glioblastoma is the mutation of the EGFR receptor, which can occur concurrently with the formation of hybrid genes (e.g., EGFR/SEPT14 and EGFR/TACC) (OMIM 131550; OMIM 612140). In other cases, a mutant variant known as EGFRvIII emerges, leading to heightened SVAT3 expression through phosphorylation (OMIM 601743). Consequently, elevated SVAT3 expression correlates with active glioblastoma progression, as does the formation of the SVAT3/OSMR coreceptor complex (OMIM 601743). The predominant mutations in the EGFR gene involve deletions of exons 2–7 and amplification (OMIM 131550), which can be detected using *in situ* hybridization. Another gene of practical significance is RAB3D, associated with decreased survival rates (OMIM 604350).

Overall, glioblastoma, like many other CNS tumors, is characterized by uncontrolled growth driven by aberrant activation of tyrosine kinases (61). Notably, glioblastoma samples have shown the presence of hybrid FIG/ROS genes, associated with constitutive tyrosine kinase activation, akin to FGFR1 gene mutation (OMIM 165020; OMIM 136350). Deletions in NFKBIA, NF1, and TRIM8 genes have also been linked to poor survival rates (OMIM 164008; OMIM 613113; OMIM 606125). Loss of heterozygosity or complete inactivation is more common in tumor suppressor genes, including WDR11, BAX, and MXI1 (OMIM 606417; OMIM 600040; OMIM 600020). Additionally, co-amplification of NIPSNAP2 and VOPP1 has been shown to accelerate tumor growth and worsen prognosis (OMIM 603004; OMIM 611915). Angiogenesis plays a crucial role in tumor growth, which, in the case of high-grade gliomas, can be stimulated by mutations in the p53 gene (OMIM 191170). In benign tumors, neo-vascularization is inhibited by the expression of ADGRB1 (OMIM 602682).

Fragments containing “oligodendroglioma/+oligodendroglioma” were discovered in 31 sources within the OMIM database. However, the majority of genes harbor mutations that are challenging to assess using *in situ* hybridization. For instance, mutations in PIK3CA have been linked to anaplastic oligodendroglioma (OMIM 171834). Oligodendrogliomas are characterized by elevated expression of OLIG1/2, which is specific to this tumor type (OMIM 606385; OMIM 606386). Similarly to glioblastoma, increased expression of the metalloproteinase MMP24 is associated with a poor prognosis (OMIM 604871). While the loss of tumor suppressor gene expression is not unique to oligodendroglioma, specific losses of DMBT1 and NKX6-2 are notable (OMIM 601969; OMIM 605955). Detection of TRIM8 is common in both glioblastomas and anaplastic oligodendrogliomas (OMIM 606125). Lastly, heightened expression of ATP8A1 and ATAD2B is observed, with the latter also detectable in glioblastoma cells (OMIM 609542; OMIM 615347).

### Meningiomas

Meningiomas are predominantly benign, comprising about 85% of cases classified as Grade I according to the WHO classification. They typically have a favorable prognosis, with a low recurrence risk not exceeding 5% (62, 63). However, it’s important to note the existence of malignant variants and instances of aggressive progression characterized by rapid growth and pronounced clinical symptoms (64).

The FISH analysis offers a distinct advantage in identifying mutations that signal a negative prognosis, even in cases lacking clear histological indicators. Among the earliest mutations often detected through FISH analysis are those occurring in the NF2 gene, present in nearly half of all meningioma cases (65). These early mutations typically include monosomy of chromosome 22, where the NF2 gene is located, as well as loss of the DAL-1 gene (66). Further malignant progression of meningiomas is associated with deletions in chromosomes 1p or 14q (67). Notably, the prognosis is considered particularly poor in cases of co-deletion of 1p/14q, even in the absence of histological signs of malignancy (64, 68, 69).

In addition to the aforementioned mutations, some researchers have identified an association between anaplastic meningioma and the amplification of the 17q23 region and PS6K, both of which are linked to tumor progression (27). A recent study revealed that grade I meningiomas recurring after resection were often associated with the deletion of the p36 region of the 1st chromosome (70). Through quantitative FISH analysis, it was found that meningiomas with a higher degree of malignancy (WHO grade III) exhibit shorter telomeres (71). Additionally, FISH analysis has indicated that deletions in the 17q region may serve as early markers of tumor progression (72). Thus, fluorescence *in situ* hybridization can be considered one of the primary approaches for molecular-genetic profiling of meningiomas.

**Figure 2:**
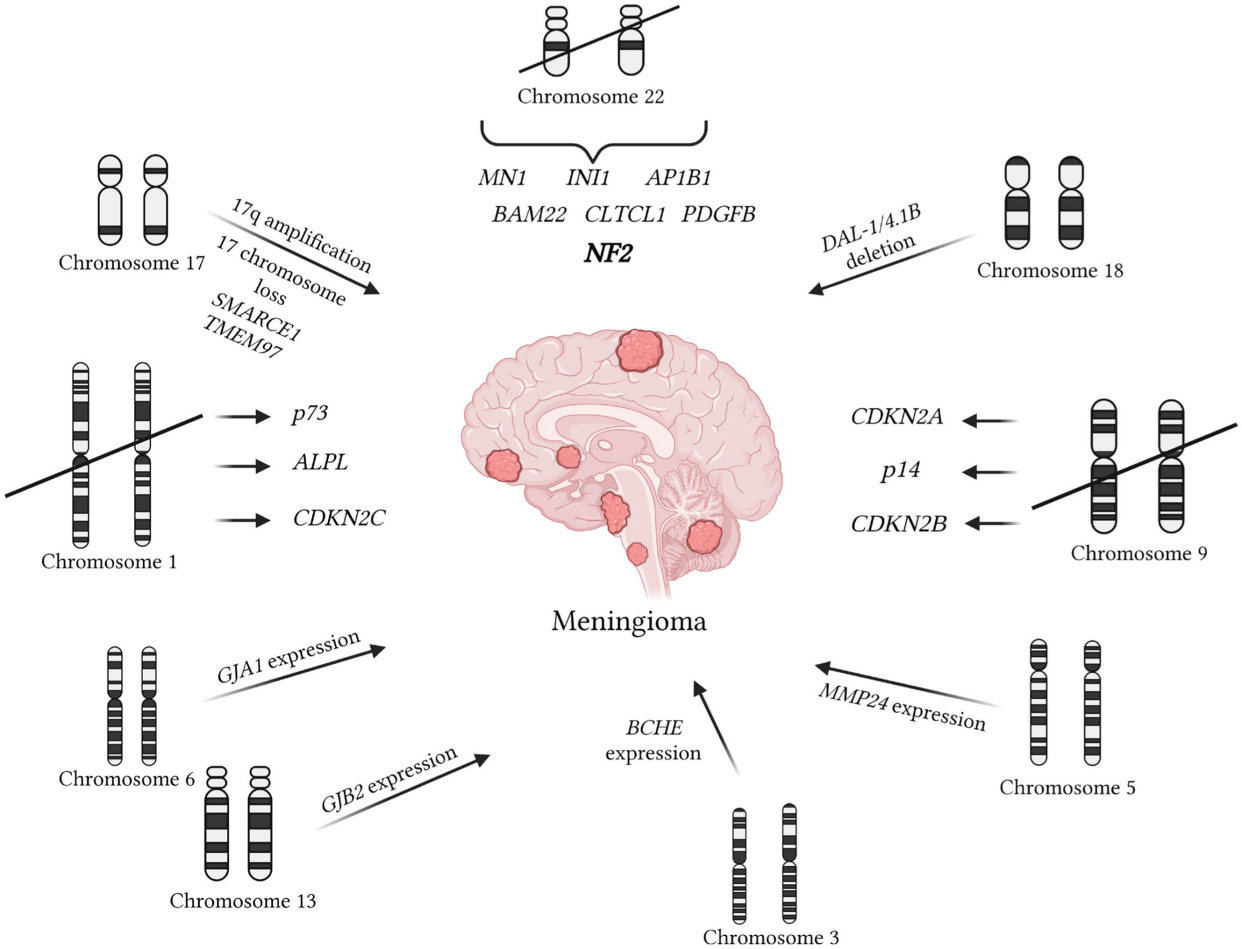
Mutations contributing to meningioma development are documented in the OMIM database. The primary mutation initiating meningioma formation is associated with the loss of chromosome 22, resulting in the absence of the NF2 gene (OMIM 607379). More aggressive forms of meningioma are linked to the loss of chromosome 1 (67). Deletions affecting the ALPL and CDKN2C genes on chromosome 1 are also associated with meningiomas (68, 69). An early event is the deletion of DAL1, which typically functions as a tumor suppressor (OMIM 605331). Additionally, the deletion of the PDGFB gene on chromosome 1, associated with early tumor development, has been noted (OMIM 190040). The SMARCE1 marker on chromosome 17 is characteristic of spinal meningiomas (OMIM 603111). Abnormalities in chromosome 17 are common, unlike chromosome 9, which plays a crucial role in tumor-suppressing genes such as CDKN2B, p14, and CDKN2A (68, 73). Furthermore, increased expression of the metalloproteinase MMP24, also present in oligodendrogliomas, has been detected (OMIM 604871). High levels of butyrylcholinesterase (BCHE), similarly found in glioblastomas and neuroblastomas, have also been identified (OMIM 177400). Elevated levels of connexins GJB2 on chromosome 13 and GJA1 on chromosome 6 have been observed (OMIM 121011; OMIM 121014). The presence of MMP25 indicates that the tumor is not a meningioma but rather an astrocytoma or glioblastoma (OMIM 608482). It is important to note that MMP25 was not present in normal brain tissue.

During the analysis of 64 entries from the OMIM database using the queries “meningioma/+meningioma,” additional mutations available for detection by *in situ* hybridization methods were identified (Figure 2). The primary mutation leading to meningioma development is often the loss of chromosome 22, resulting in the loss of the NF2 gene, which inhibits tumor formation (OMIM 607379). formation (OMIM 607379). Besides mutations in this gene, other regions of chromosome 22 associated with meningiomas were also identified. It is believed that in the presence of an intact chromosome 22, the loss of the first chromosome is necessary for the development of a more aggressive anaplastic variant of meningioma (73). Independent deletions of the ALPL and CDKN2C genes, located on the first chromosome, were detected in patients with meningiomas (74, 75). Regarding the involvement of p73 in meningioma development, available information does not indicate correlations between clinical outcomes and expression (76). However, some studies suggest an increase in p73 expression with tumor grade (77). Another early event considered is the deletion of DAL1, which acts as a tumor suppressor under physiological conditions (OMIM 605331).

It’s worth highlighting the deletion of the PDGFB gene located on chromosome 1, which is associated with early tumor onset (OMIM 190040). Another notable marker specific to spinal meningiomas is the region on chromosome 17, SMARCE1 (OMIM 603111). Chromosome 17 abnormalities, such as deletions and amplifications, are often observed in meningiomas, unlike chromosome 9, which is almost always lost in meningioma cells (78). The deletion of chromosome 9 is linked to the loss of important genes CDKN2B, p14, and CDKN2A, which normally suppress tumor development (74, 79). Mutations in the IGFBP7 signaling pathway, also disrupted in various carcinomas, are identified in meningiomas (OMIM 602867). Like other central nervous system tumors, meningiomas can develop as part of tumors with multiple localizations. For example, this occurs with the loss of heterozygosity of the BAP-1 gene (OMIM 603089). Additionally, there’s an intriguing increase in the expression of the TNKS2 gene in meningioma cells, potentially associated with the immune response to tumor development (OMIM 607128).

Additionally, in the analysis of mutations in meningioma cells, heightened expression of metalloproteinase MMP24 was observed, a feature also present in oligodendrogliomas (OMIM 604871). Furthermore, the presence of overexpressed MMP25 suggests the likelihood of the tumor being an astrocytoma or glioblastoma (OMIM 608482). High levels of cholinesterase BCHE were also detected, a characteristic shared with glioblastomas and neuroblastomas (OMIM 177400). Lastly, elevated expression levels of connexin GJB2 on chromosome 13 and GJA1 on chromosome 6 were identified (OMIM 121011; OMIM 121014).

### Ependymomas

Ependymoma, more frequently encountered in young individuals and children, is associated with neurofibromatosis type II (80). Therefore, primary investigations, including FISH analysis, are focused on the q12 region of chromosome 22, where the NF2 gene is localized (80). Many researchers note that histological classification alone is insufficient for accurate disease prognosis, and ependymoma itself carries a poor prognosis (81). It has become evident that relying solely on FISH analysis is inadequate for identifying potential disease prognosis markers (82). However, research findings using this method have identified amplification of chromosome 1, which correlates with a high degree of tumor malignancy. For instance, amplification of regions 1q21.1–32.1 has been associated with tumor recurrence, while amplification of 1q25 serves as an independent prognostic marker for patient survival (76). Recently, a specific gene translocation involving RELA and C11orf95 has been discovered, leading to the formation of a new oncogene (77).

The search using the keywords “ependymoma/+ependymoma” revealed 21 results in the OMIM database. The predominant mutation found in most ependymomas involves the formation of the oncogene C11ORF95-RELA. This fusion gene can migrate into the nucleus, activating NF-κB and promoting tumor growth (OMIM 615699). Additionally, ependymomas exhibit amplification of EPHB2 and high expression of CIZ1 (OMIM 600997; OMIM 611420). Furthermore, noteworthy is the expression of the H2-delta haplotype of the PDGFRA gene, which is also present in many embryonal tumors (OMIM 173490).

### Medulloblastoma and embryonal tumors

Previously, medulloblastomas were classified into subgroups based on ErbB2 expression levels measured via immunohistochemistry (83). Elevated ErbB2 expression has been associated with the loss of the short arm of chromosome 17 and amplification of the long arm (84). Rare chromosomal anomalies, such as amplification of the myc oncogene (occurring in 6% of cases), have been linked to unfavorable prognoses (85). Despite the low frequency of oncogene amplification across different medulloblastoma subtypes, recent studies highlight the prognostic significance of detecting C-myc/N-myc amplification at the single-cell level using FISH analysis (86). Deletions on chromosomes 10q, 16q, and 8p, as well as amplifications of chromosomes 2, 7, and 17, have also been identified in medulloblastomas (87). Notably, p53 gene mutations associated with medulloblastoma recurrences are detected in almost all central nervous system tumors (88). Molecular genetic methods have allowed the classification of medulloblastomas into four groups (89). The group with WNT gene mutations is characterized by a favorable prognosis, unlike groups 3 and 4. The presence of isochromosome 17q serves as an important prognostic marker, determined in part using fluorescence *in situ* hybridization (85).

Medulloblastomas exhibit histological similarities to embryonal tumors, particularly primitive neuroectodermal tumors (90). One key criterion for distinguishing medulloblastoma is the detection of isochromosome 17q and the presence of chromosomes 14q and 19q, whose deletion is characteristic of primitive neuroectodermal tumors (91, 92). Rhabdoid tumors (highly malignant embryonal tumors) are characterized by monosomy of chromosome 22 or a mutation in the hSNF5/INI1 gene located on the same chromosome (93, 94).

**Figure 3:**
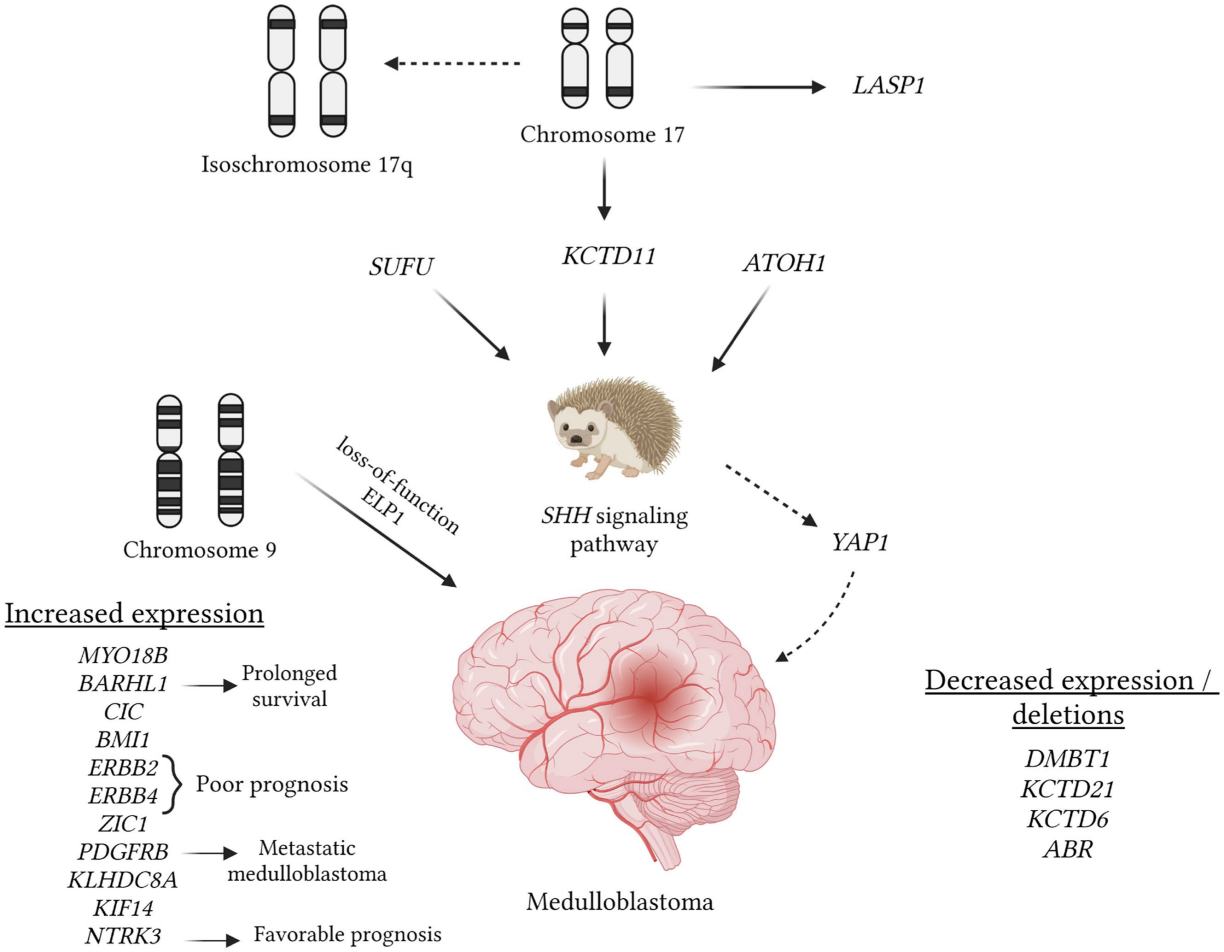
Understanding the role of various mutations in medulloblastoma pathogenesis, which can be identified using *in situ* hybridization methods, is crucial. The figure provides an overview of mutations cataloged in the OMIM database that are linked to medulloblastomas. In this tumor type, a pivotal event involves the hyperactivation of the SHH pathway, potentially due to mutations in the SUFU gene, loss of the 17th chromosome (resulting in the loss of the KCTD11 potassium channel gene), and the expression of the ATOH1 gene. The latter inhibits neuronal differentiation, thereby promoting medulloblastoma progression (OMIM 609848; OMIM 618790; OMIM 601461). Isochromosome 17q is a commonly observed aberration visualized through hybridization techniques. Conversely, amplification of the 17q region may lead to increased expression of LASP1, thereby stimulating tumor cell proliferation (OMIM 602920). Loss of the 9q chromosome segment is associated with additional loss of function in the ELP1 gene, potentially predisposing individuals to medulloblastoma development (OMIM 603722). Enhanced expression of ERBB2, ERBB4, and PDGFRB correlates with an unfavorable prognosis and tumor metastasis (OMIM 164870; OMIM 600543; OMIM 173410). In contrast, the expression of BARHL1 and NTRK3 is associated with a favorable prognosis and longer intervals without tumor progression (OMIM 60524; OMIM 191316). Besides KCTD11, deletions involving KCTD21 and KCTD6 may also occur (OMIM 618790; OMIM 618791). Similarly to many other tumors, the deletion of the DMBT1 gene is noted (OMIM 601969).

The analysis of 107 entries in the OMIM database using the keywords “medulloblastoma/+medulloblastoma” (Figure 3) revealed pivotal stages in pathogenesis, notably the disruption of the SHH signaling pathway (OMIM 600725). Mutations in the SUFU gene are recognized as one of the factors contributing to pathway hyperactivation and are occasionally associated with meningioma development (OMIM 607035). Alterations in the SHH gene are frequently linked to the desmoplastic subtype of medulloblastoma (OMIM 600725). Conversely, deletions in the ATOH1 gene inhibit the SHH signaling pathway, thwarting medulloblastoma development (OMIM 601461). Anomalies in this pathway can also induce other changes, such as increased expression of YAP1 (OMIM 606608).

Under normal circumstances, the expression of the KCTD11 gene on the 17th chromosome can inhibit SHH, and this gene is frequently subject to deletion in cases of medulloblastoma (OMIM 609848). Additionally, deletions of KCTD21 and KCTD6 may occur (OMIM 618790; OMIM 618791). As observed in many other tumors, deletion of the DMBT1 gene is also noted (OMIM 601969) in medulloblastoma. Furthermore, medulloblastoma entails a frameshift mutation in the GPR161 gene, which encodes one of the types of G protein-coupled receptors (OMIM 612250). Employing *in situ* hybridization methods during the investigation of medulloblastoma can prove valuable in conducting NGS, facilitating a more precise selection of specific DNA regions, and enabling the comparison of mutation sites with the wild type.

There are medulloblastoma variants characterized by amplification of the 17q chromosome, resulting in heightened expression of the LASP1 gene (OMIM 602920). Research indicates that suppressing this gene significantly reduces cell proliferation in medulloblastoma (OMIM 602920). Loss of the 9q chromosome segment is linked to additional loss of function in the ELP1 gene, potentially predisposing individuals to medulloblastoma development (OMIM 603722). Moreover, medulloblastoma exhibits increased expression of genes such as MYO18B (often absent in other tumors), ERBB2, ERBB4, BMI1, and KLHDC8A (OMIM 607295; OMIM 164831; OMIM 614503; OMIM 155255; OMIM 164870; OMIM 600543). Notably, the detection of ERBB2, ERBB4, and PDGFRB expression, which correlates with an unfavorable prognosis and metastasis, does not always indicate tumor progression (OMIM 155255; OMIM 173410). Conversely, overexpression of BARHL1 and NTRK3 has been associated with longer remission intervals and a more favorable prognosis (OMIM 60524; OMIM 191316).

### Craniopharyngioma

This tumor type often displays aggressive behavior, impacting adjacent brain structures (95, 96). The adamantinomatous subtype occurs uniformly across age groups and is marked by specific mutations in the CTNNB1 gene, responsible for encoding beta-catenin (97). These mutations trigger the accumulation of mutated beta-catenin, thereby activating the Wnt signaling pathway, pivotal in tumor development. Conversely, the papillary subtype, more prevalent in adults, is defined by the BRAF V600E mutation (98, 99). Due to the rarity of this tumor variant and the focus on more aggressive processes, research efforts often remain constrained to observational accounts of these mutations. A study in 2022 utilized FISH analysis to reveal heightened expression of SERPINE1+ and SERPINEG1+ in macrophages surrounding adamantinomatous tumors (100). In another investigation, increased expression of the tyrosine kinase TrkA was identified. However, this analysis employed a combination of methods including immunohistochemistry, PCR, and FISH, with the latter specifically targeting NTRK1 fusions (101).

The OMIM database contains only 3 entries containing the keywords “craniopharyngioma/+craniopharyngioma.” Among them, the ACVR1 gene was identified, which is highly impractical to detect using *in situ* hybridization due to the specificity of the mutation, characterized by the replacement of arginine with histidine at codon 20 (OMIM 102576).

### Adenomas and adenocarcinomas

Adenomas and adenocarcinomas of the pituitary gland encompass a wide spectrum of tumors, classified into functional (often microadenomas) and non-functional (typically macroadenomas) categories (102). Notably, among the various adenoma types, some exhibit high invasiveness, such as non-functional corticotropin and thyrotropin adenomas (103, 104). Tumors with high invasiveness, based on molecular-genetic characteristics, often mimic carcinomas, adding significant interest for research purposes (104–106). Many researchers advocate for combining FISH analysis with cytogenetic studies in comparative genomic hybridization, presenting a promising avenue for investigation (107). Additionally, the majority of identified genetic alterations in adenoma development have been elucidated using immunohistochemical methods (107, 108).

A method proposed some time ago assesses the percentage of positively stained nuclei in tumor samples using the Ki-67 marker, where a level exceeding 3% indicates potential tumor invasiveness (109). It’s worth noting that diminished expression of the tumor suppressor p27 is linked to invasive forms of pituitary adenomas and carcinomas (110). Comparative genomic hybridization has unveiled a significant number of chromosomal anomalies, most frequently encountered in tumors producing prolactin and growth hormone. Examples of such anomalies include monosomy of chromosome 11, trisomies of chromosomes 8 and 12 (111).

**Figure 4:**
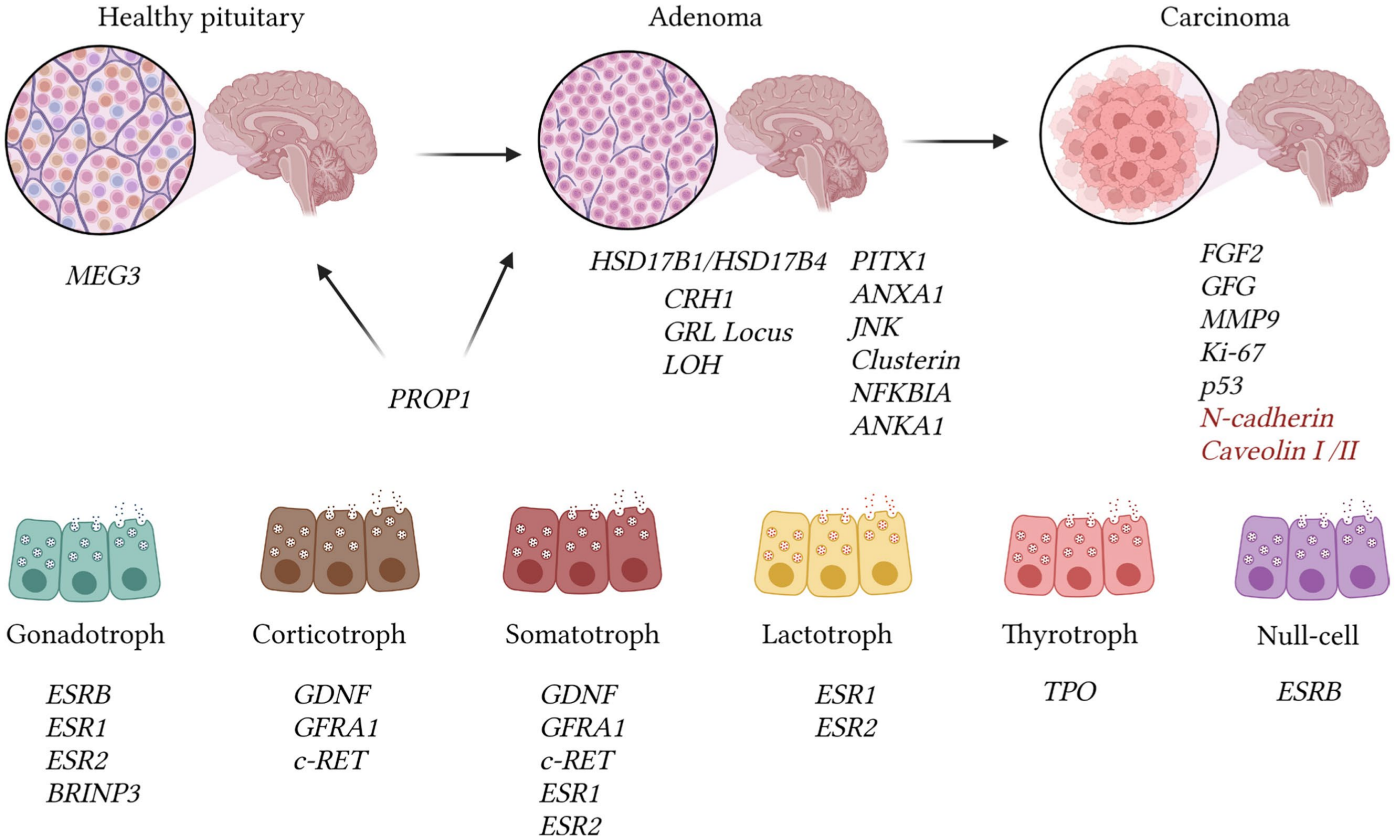
The illustration presents genetic changes identified in pituitary adenomas, drawing on data from the OMIM database and other reputable sources. Adenomas exhibit a reduction in the expression of several genes, including JNK, Clusterin, NFKBIA, ANKA1, and PITX1 [OMIM 602149; (107)]. The transition to pituitary carcinomas correlates with increased invasiveness, as reflected in alterations in MMP9 expression (OMIM 120361). Pituitary carcinomas demonstrate heightened expression of FGF2 and diminished GFG mRNA levels. Assessment of malignancy encompasses Ki-67 index (>10%) and p53 expression intensity. The expression of N-cadherin and decreased caveolins I/II levels are linked to metastasis. Nevertheless, such changes may also manifest in ordinary adenomas, according to recent research findings, hence we highlighted them in red (107). The MEG3 gene functions as a suppressor of adenoma development and is exclusively present in normal pituitary tissue (OMIM 605636). The PROP1 gene is identifiable in adenomas (OMIM 601538). Expression of ESRB is distinctive in gonadotroph and null-cell adenomas, whereas ESR1 and ESR2 mRNAs are concurrently expressed in prolactinomas, somatotropinomas, and prolactinomas (107–110). GDNF, GFRA1, and c-RET are detected in corticotroph and somatotroph tumors. Gonadotrophic tumors are characterized by BRINP3 gene expression, which is associated with tumor proliferation and invasion [OMIM 618390; (107–109)].

The OMIM database contains 53 entries with the keywords “pituitary adenoma,” but most of the gene mutations are challenging to verify using hybridization methods (Figure 4). Mutations that can be visualized using fluorescent or comparative hybridization include increased expression of CRHR1 or the C-RET gene, primarily among GH and ACTH-secreting adenomas (OMIM 122561; OMIM 164761). GDNF expression is more characteristic of GH and corticotroph adenomas, while GFRA1 expression is associated with corticotroph and somatotroph tumors (OMIM 600837; OMIM 601496). Co-expression of ESR1 and ESR2 mRNA was observed in prolactinomas as well as somatotroph and gonadotroph tumors (OMIM 601663; OMIM 133430). Interestingly, MMP9 and FGF2 expression was characteristic of invasive adenomas and pituitary carcinomas (OMIM 120361) (112). Moreover, higher expression of FGF2 relative to GFG was associated with more aggressive tumor behavior (113). The presence of BRINP3 expression was linked to gonadotroph adenomas, with this gene believed to induce proliferation, migration, and further invasion of the tumor (OMIM 618390).

In adenomas, there is a notable decrease in the expression of several genes, including JNK, Clusterin, NFKBIA, ANKA1, and PITX1 [OMIM 602149; (113)]. Loss of heterozygosity at the GRL locus may contribute to tumor resistance to negative feedback, while the expression of N-cadherin and reduced levels of caveolins I/II are associated with metastasis (113). It has been determined that the MEG3 gene acts as a suppressor of adenoma development and is exclusively present in normal pituitary tissue (OMIM 605636), whereas the PROP1 gene is detected in adenomas (OMIM 601538). Expression of ESRB is distinctive in gonadotroph and null-cell adenomas, while genes ESR1 and ESR2 are co-expressed in prolactinomas, somatotropinomas, and prolactinomas (113–115).

There is particular interest in examining the expression of various genes in hormone-producing adenomas. For example, genes HSD17B1 and HSD17B4 were found to be expressed across all types of adenomas (OMIM 109684; OMIM 601860). HSD17B3 expression was ubiquitous except in corticotroph adenomas, while HSD17B2 was present in all types except prolactinomas (OMIM 605573; OMIM 109685). Additionally, the growth suppressor MEG3 was identified, exhibiting exclusive expression in non-tumorous gonadotrophs (OMIM 605636). PPAR-gamma expression was observed in ACTH-producing tumors, and the estrogen receptor isoform ESRB was characteristic of null-cell and gonadotroph adenomas (116).

Currently, mutations in the PTTG gene have been identified, which are characteristic of functional pituitary adenomas, alongside mutations in the BRAF and MEN1A genes, potentially responsible for sporadic occurrences of these adenomas (117, 118). New mechanisms of pituitary adenoma pathogenesis have been proposed, including the amplification of HMGA2 (118). This process likely occurs through acetylation, enhancing the activity of E2F1 (119). Recently, telomeres in pituitary tumors were assessed at the single-cell level using FISH analysis (120). The findings revealed that telomere shortening and alternative lengthening (independent of telomerase) were associated with invasive carcinomas. The data indicated that 59.4% of samples exhibited shortened telomeres, while the presence of alternative telomere lengthening correlated with tumor recurrence.

**Figure 5:**
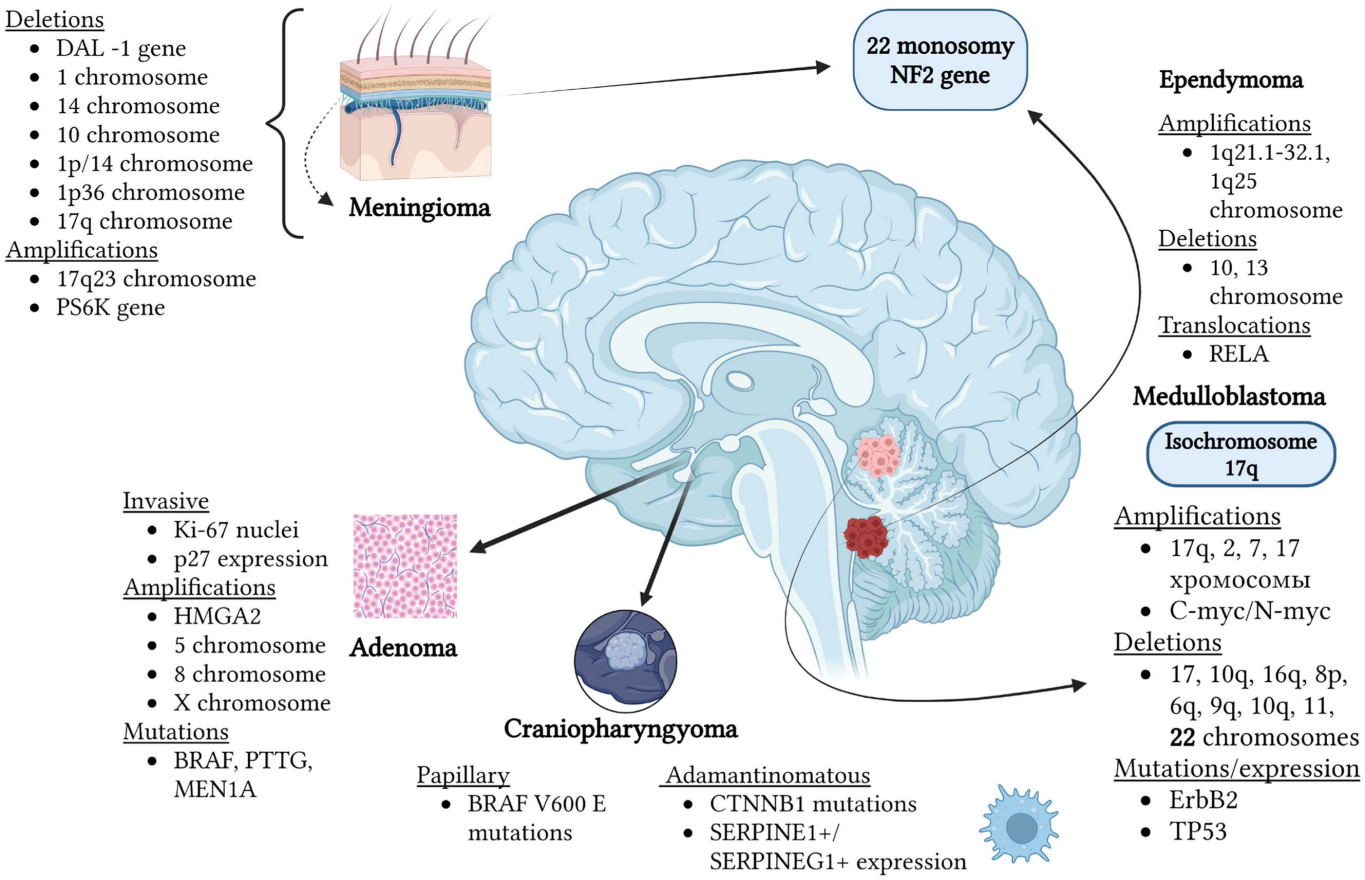
Mutations and chromosomal anomalies are detected in various types of brain tumors through FISH hybridization. As depicted in the figure, each tumor exhibits distinct genetic and chromosomal anomalies occurring within cells. The commonality between ependymomas and meningiomas (rarely for medulloblastomas) is monosomy of chromosome 22, alongside NF2 gene mutations located on this chromosome. Hence, the listed tumors may manifest within the spectrum of neurofibromatosis type 2. While some identified anomalies directly contribute to tumor development, others serve as prognostic markers. For instance, the development of pituitary adenomas has been associated with E2F1 acetylation and subsequent HMGA2 gene amplification. Overall, adenomas display extremely high heterogeneity depending, for example, on the hormone they produce. Prolactin-producing tumors are characterized by monosomy of chromosome 11 and trisomies of chromosomes 8 and 12. Amplification of chromosomes 5, 8, and X is typical for pituitary adenomas. Meningiomas with the presence of 17q23, PS6K amplifications, and 1p/14 co-deletions indicate the worst prognosis, while 1p36 deletion suggests a high likelihood of recurrence. In ependymomas, a survival marker can be the amplification of the 1q25 chromosome region. Additionally, in some cases, monosomies of chromosomes 10 and 13 are observed. Notably, the presence of isochromosome 17q in medulloblastomas allows differentiation from other embryonal tumors also developing in the cerebellum. The mutations listed in the figure can be identified through hybridization methods but do not represent a comprehensive list of all gene anomalies characteristic of different types of brain tumors (62–64, 74–77, 80–82, 103, 104, 107, 111, 112, 121, 122).

Finally, we have synthesized chromosomal anomalies and specific mutations uncovered through literature scrutiny, independent of the OMIM database. These findings are depicted in Figure 5, excluding glioblastomas and oligodendrogliomas, which receive more comprehensive treatment in the table and Figure 1 (123–130). It’s apparent that each tumor showcases distinct genetic and chromosomal irregularities detected within cells. The sole common thread linking ependymomas and meningiomas (occasionally, medulloblastomas) is the presence of monosomy 22 chromosome, alongside mutations in the NF2 gene located on this chromosome. Thus, the enumerated tumors may manifest within the spectrum of neurofibromatosis type 2. While some identified anomalies play a direct role in the tumor’s pathogenesis, others function as prognostic indicators.

## Conclusion

Molecular-genetic diagnostics of the most common and malignant brain tumors encompasses a wide range of genetic and chromosomal anomalies. The application of *in situ* hybridization methods, including their combination with PCR, sequencing, and cytogenetics, holds significant potential in diagnosing and prognosticating tumors such as glioblastomas, oligodendrogliomas, meningiomas, ependymomas, medulloblastomas, pituitary adenomas, and adenocarcinomas. Currently, the FISH hybridization method is primary in this field, but there are modifications that can improve outcomes. While some chromosomal changes (e.g., chromosomes 1, 10, 17, and 22) and genetic mutations (e.g., MMP25, MMP9, NFKBIA, and DMBT1) are characteristic of several types of brain tumors, detecting changes specific to certain tumors (e.g., the formation of isochromosome 17q in medulloblastoma) is crucial for prognosis and therapy effectiveness. However, despite the significance of *in situ* hybridization, most mutations identified in the OMIM database require PCR or sequencing for identification, which currently does not allow *in situ* hybridization to be recognized as the primary method for diagnosing brain tumors.

## Author contributions

EN: Conceptualization, Data curation, Writing – original draft, Writing – review & editing. GZ: Conceptualization, Data curation, Visualization, Writing – original draft, Writing – review & editing. PT: Conceptualization, Data curation, Supervision, Writing – original draft, Writing – review & editing.
